# Diurnal change of retinal vessel density and mean ocular perfusion pressure in patients with open-angle glaucoma

**DOI:** 10.1371/journal.pone.0215684

**Published:** 2019-04-26

**Authors:** Sung Uk Baek, Young Kook Kim, Ahnul Ha, Yong Woo Kim, Jinho Lee, Jin-Soo Kim, Jin Wook Jeoung, Ki Ho Park

**Affiliations:** 1 Department of Ophthalmology, Hallym University College of Medicine, Anyang, Korea; 2 Department of Ophthalmology, Hallym University Sacred Heart Hospital, Anyang, Korea; 3 Department of Ophthalmology, Seoul National University College of Medicine, Seoul, Korea; 4 Department of Ophthalmology, Seoul National University Hospital, Seoul, Korea; Bascom Palmer Eye Institute, UNITED STATES

## Abstract

**Purpose:**

To investigate, in primary open-angle glaucoma (POAG) and healthy subjects, the pattern and magnitude of diurnal variation in optical coherence tomography angiography (OCTA) retinal vessel density (RVD).

**Design:**

Prospective, observational cross-sectional study.

**Participants:**

A prospective study was conducted on 20POAG patients and 19 healthy subjects.

**Methods:**

Peripapillary/macular RVD (using swept-source OCTA), intraocular pressure (IOP), and systemic blood pressure (BP) were measured five times a day (8 a.m., 11 a.m., 2 p.m.,5 p.m. and 8 p.m.). The magnitudes and patterns of diurnal changes in RVD, diastolic BP, and mean ocular-perfusion pressure (MOPP) were analyzed and compared between the POAG patients and the healthy subjects.

**Main outcome measures:**

The patterns and magnitudes of diurnal RVD change in OCTA.

**Results:**

Intra-visit repeatability (0.755–0.943) and inter-visit reproducibility (0.843–0.986) for the RVD measurements showed excellent reliability. In the POAG patients, the magnitude of diurnal change in peripapillary RVD (9.71±7.04%) and macular RVD (7.22±4.73%) were significantly greater than that in the healthy group (5.73±3.85%, *P* = 0.013 and 5.51±3.45%, *P* = 0.042, respectively). The magnitudes of diurnal variations of IOP and MOPP in the POAG group likewise were greater than those in the healthy group (*P* = 0.003 and 0.039). As for the patterns of diurnal RVD change, interestingly, at 8 p.m., the macular RVD of the healthy group increased to the highest level (44.12±2.95%) while that of the POAG group decreased to the lowest level (40.41±2.54%).

**Conclusions:**

In POAG eyes, diurnal change of IOP, MOPP and RVD was significantly greater than in the healthy eyes. These findings suggest that diurnal RVD changes might reflect the hemodynamic variation of POAG.

## Introduction

In glaucoma, impaired microcirculation at the level of the optic nerve head (ONH) has been suggested to be one of the main mechanisms of pathogenesis [1–3]. In this regard, optical coherence tomography angiography (OCTA) can be a helpful tool to understand the vulnerability of retinal microcirculation in glaucoma. OCTA is a relatively new, non-invasive imaging technique that uses flow-based information to directly visualize the perfused retinal vasculature by means of motion contrast. Retinal blood flow is determined by analysis of repeated scans obtained at an identical position of the retina [4–6].

Several OCTA studies have reported reduced blood flow in the ONH of glaucoma patients compared with normal individuals [6]. Specifically, the density of the peripapillary vasculature is significantly lower in glaucoma than in normal eyes, and is well-correlated with the functional and structural severity of glaucoma [7–10]. However, these studies were cross-sectional and did not fully investigate the related factors.

Retinal vessel density (RVD) could be related to ocular-perfusion pressure [6], which is determined by intraocular pressure (IOP) and blood pressure (BP) [11, 12]. Meanwhile, mean ocular-perfusion pressure (MOPP) has shown greater magnitudes of diurnal variation in glaucoma patients than in normal individuals [13, 14]. Therefore, we can expect that RVD also might show diurnal variation and that the pattern might differ between glaucoma patients and normal individuals. However, there is a lack of research into the diurnal variation of RVD through OCTA in glaucoma patients [15].

Thus prompted, the present study aimed to examine the diurnal variation of RVD and the related oculo- and hemo-dynamic factors including IOP, BP, and MOPP between primary open-angle glaucoma (POAG) patients and normal subjects.

## Methods

The study protocol was approved by the Institutional Review Board of Seoul National University Hospital and adhered to the tenets of the Declaration of Helsinki. Each subject was required to sign an informed consent statement before being enrolled into the study and prior to any study measurements being taken.

### Study subjects

This was a prospective cohort study of POAG and age-matched healthy subjects. All of the subjects were prospectively enrolled at the Glaucoma Clinic of Seoul National University Hospital between May and November 2017.

All of the participants had to meet the following criteria for inclusion: greater than 18 years of age, best-corrected visual acuity (BCVA) ≥ 20/40, open angle status on gonioscopic examination, and spherical equivalent less than -6.0 diopters. Patients with a history of ocular surgery other than uncomplicated cataract surgery, or any history of retinal pathology, or non-glaucomatous optic neuropathy, were excluded. Participants also were excluded in cases of diagnosis of dementia or Alzheimer’s or stroke history. Participants with systemic hypertension and diabetes mellitus were included unless they had been diagnosed with diabetic or hypertensive retinopathy. Existing medication, including topical antiglaucoma therapy and systemic antihypertensive drugs, was continued throughout the study. However, patients on oral carbonic anhydrase inhibitors were excluded [16]. After evaluating the enrollment of glaucoma patients, we then consecutively recruited healthy volunteers from a comprehensive ophthalmology clinic in consideration of the age distribution of glaucoma patients. Healthy subjects were assigned to age groups by decade (e.g., a ‘36 years old’ subject was classified as ‘30s’).

All of the participants underwent complete ophthalmologic examinations, including IOP measurement by Goldman applanation tonometry at the baseline exam, BCVA, refraction with an autorefractor (KR-890; Topcon Corporation, Tokyo, Japan), slit-lamp examination, gonioscopy, and dilated fundus examination. After maximum pupil dilation, all of the participants were imaged by stereo optic disc photography and red-free RNFL (retinal nerve fiber layer defect) photography (Vx-10; Kowa Optimed Inc., Tokyo, Japan). They also underwent standard automated perimetry using the Swedish interactive threshold algorithm with the 30–2 standard program (Humphrey Field Analyzer II; Carl Zeiss Meditec Inc).

Glaucomatous eyes were defined as those showing glaucomatous optic disc neuropathy (e.g., notching, neuroretinal rim thinning, and/or RNFL defects) and corresponding glaucomatous visual field defects, as confirmed by at least two consecutive visual field examinations. Glaucomatous visual field defects were defined as a cluster of ≥ 3 points with P < 0.05 on the pattern deviation map in at least one hemifield, including ≥ 1 point with P < 0.01; a pattern standard deviation of P < 0.05; or glaucoma hemifield test result outside the normal limits. The criteria for inclusion in the age-matched healthy group were IOP < 21 mm Hg with no history of elevated IOP, a normal anterior segment on slit-lamp examination, no RNFL defects in red-free RNFL photographs, and no glaucomatous visual field defects within 6 months of OCTA imaging. A normal visual field was defined as a pattern standard deviation within the 95% confidence limits and a Glaucoma Hemifield Test within normal limits. To eliminate individual variations, only one eye, mostly the right eye, was examined for each subject. However, we studied the left eye if the right met any of our exclusion criteria.

### Measurement of diurnal variation of RVD by swept-source optical coherence tomography angiography (SS-OCTA) measurements

The OCTA scans were performed at five different time points over a single day at 3-hour intervals: 8:00 a.m., 11:00 a.m., 2:00 p.m., 5:00 p.m., and 8:00 p.m. All of the OCTA scans were performed by the same experienced OCTA operator (S.U.B.) under pupil dilation. Patients were instructed to avoid caffeine intake, smoking, and exercise for 3 hours prior to the study visit.

**Fig 1:**
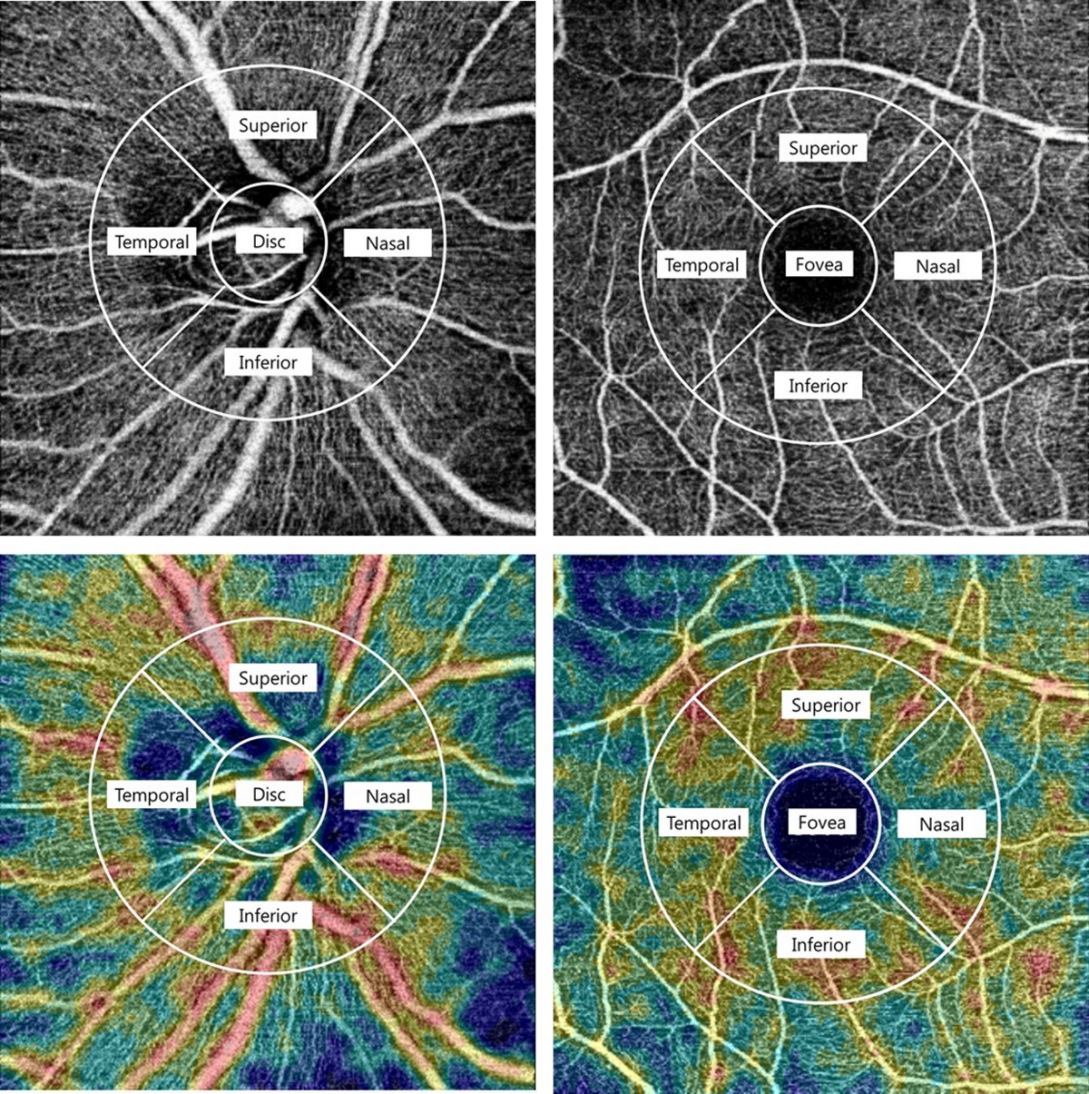
Swept-source optical coherent tomography angiography (SS-OCTA) scan and of the peripapillary area and macula showing superficial vascular plexus. Swept-source optical coherent tomography (SS-OCT) scan (4.5 x 4.5 mm) of the peripapillary area and macula showing superficial vascular plexus as entire en face region (top). The value of the retinal vessel density (RVD) was calculated based on the intensity of the OCTA image. The “Density Map” (bottom) is a color map generated according to the brightness averages of the OCTA images.

In the present study, we applied a mode of swept-source optical coherence tomography angiography (SS-OCTA) (DRI OCT Triton, Topcon, Tokyo, Japan) to visualize the vasculature of the ONH and macula (Fig 1). The device operates with a central wavelength of 1050 nm, an acquisition speed of 100,000 A-scans per second, and axial and transverse resolutions of 7 and 20μm, respectively, in tissue. The scans were taken from a 4.5 × 4.5 mm cube, each cube consisting of 320 clusters of four repeated B-scans centered on the fovea. The DRI OCT Triton characterizes vascular information at various user-defined retinal layers as a vessel density map and quantitatively as vessel density (%). RVD was automatically calculated as the proportion of the measured area occupied by flowing blood vessels defined as pixels having above-threshold-level decorrelation values acquired by the OCTARA (OCTA Ratio Analysis) algorithm [17].

En face images were generated from the superficial retinal layer in the macula and ONH based on automated layer segmentation performed by the OCT instrument software (IMAGEnet 6 V.1.14.8538). The automated segmentation defines the en face slab for the superficial retinal layer as extending from 2.6 μm beneath the internal limiting membrane to 15.6 μm beneath the interface of the inner plexiform layer and inner nuclear layer (IPL/INL). For this report, we analyzed peripapillary RNFL and parafoveal RNFL vessel densities in images with a 4.5 x 4.5-mm field of view centered on the optic disc and fovea.

The value of RVD was calculated based on the intensity of the OCTA image of the entire enface region (Fig 1). Peripapillary and macular RVD were measured at the optic disc and fovea and at each of the adjacent four sites employing a modified version of the Early Treatment Diabetic Retinopathy Study (ETDRS) grid. The modified ETDRS grid consists of two circles; the outer square has a diameter of 3.0 mm and the inner circle, 1.0 mm, from the centers of the disc and fovea, respectively. Moreover, in the present study, a central inner circle (0.785 mm^2^) was defined as the optic disc and fovea, and each parafoveal and circumpapillary region was defined as a ring surrounding the circular sector. Also, the parafoveal and circumpapillary regions were divided into 4 sectors, superior, inferior, temporal and nasal, and their vessel densities were calculated according to a 2.0mm-wide annulus extending from the optic disc and fovea boundary (1.571 mm^2^, respectively). A “Density Map,” which is a color map generated based on the average brightnesses of OCTA images, also was provided with en face images.

The quality of each scan and the accuracy of the segmentation algorithm were determined by masked reviewers (S.U.B. and Y.K.K.). Only images with a signal strength of ≥ 45 without segmentation failure were selected; poor-clarity images, which is to say, those with motion artifacts or localized artifacts such as floaters were discarded.

### Measurements of oculo- and hemo-dynamic parameters

IOP and BP were measured five times, every 3 hours from 8:00 a.m. to 8:00 p.m. That is, there were five measurement sessions per day, and SS-OCTA, IOP, and BP were measured respectively for each session. In order of consent to participate in the study, predetermined random sequence were applied to SS-OCTA, IOP, and BP in turn. To minimize the residual effect of accommodation or pupillary dilation during the examination, each test interval was varied by more than 5 minutes.

The IOPs of the recruited eyes were measured by rebound tonometry (Icare PRO; Finland Oy, Helsinki, Finland) in every session. All of the subjects were in a sitting position and told to relax and look straight ahead at a specific point while the tonometer was brought near the eye. In each session, six consecutive measurements were performed, the mean of which was displayed, and after which, the average of each set was recorded. One investigator (S.U.B.) performed all of the IOP measurements. The systemic BP including systolic (SBP) and diastolic (DBP) were measured on the upper left arm with an automated sphygmomanometer (OMRON HBP-1300, OMRON Healthcare [China] Co., Ltd.) while the patient was asked to remain calm in a sitting position for at least 5 minutes. Patients were instructed to avoid caffeine intake, smoking, and exercise for 3 hours prior to the study visit.

Using the measured IOP and BP data for each session, the mean arterial pressure (MAP) and MOPP were analyzed. The MAPs were calculated as follows: MAP = DBP + [1⁄3 × (SBP − DBP)] [18, 19]. It was thus possible to calculate the MOPP at any specified time from the difference between the MAP and the IOP (substituted for venous pressure) as follows: MOPP = 2⁄3 × (MAP − IOP).

### Repeatability and reproducibility

The authors deduced that the critical point of consideration was whether the RVD fluctuations measured by the method designed in this study were true diurnal variations or mere reflections of test-retest variability. Thus, the repeatability and reproducibility of RVD in the macula and peripapillary areas were analyzed prior to identifying the diurnal variability.[20]

Repeatability and reproducibility of RVD were evaluated for the first 10 individuals of each group who agreed to participate in the study. The intra-visit repeatabilities of the peripapillary and macular RVD were calculated from a subset of 10 healthy (10 eyes) and 10 POAG subjects (10 eyes), three sets of scans having been performed within a single visit. The scan sets were all obtained within 30-60s of each other. The intraclass correlation coefficients (ICCs) were used to evaluate test-retest variability [21]. The ICC was calculated by comparing three measurements obtained at the same location by a single operator. The inter-visit reproducibility of the peripapillary and macular RVDs were calculated from the same healthy and POAG subsets. The ICCs were obtained from three sets of scans performed on three separate visits [21]. Three OCT scans were performed within 30 minutes on 3 different days, for example 9:00 a.m., 9:10 a.m,. and 9:25 a.m., respectively.

### Statistical analysis

The sample size was determined by referring to existing studies [22–24]. The sample-size calculation was based on the assumption of a 10% difference in variation of RVD between POAG and healthy groups with a 1-to-1 ratio of glaucomatous to healthy eyes. For a power of 90%, the required sample size was determined to be 25 individuals per group.

ICCs using two-way random model were used to evaluate test-retest variability. ICC values can range from 0 to 1, a higher value indicating better reliability. ICCs less than 0.40 are considered poor, those between 0.4 and 0.6 fair, those between 0.61 and 0.8 good, and those greater than 0.8, excellent [25]. The repeated-measures analysis of variance (ANOVA) was used to evaluate diurnal changes in RVD, IOP, BP, and MOPP in the two groups. Additionally, in order to investigate the differences in diurnal changes of RVD between the two groups, we performed a linear mixed model with adjusted ophthalmological and systemic factors followed by post hoc testing using the independent t-test. Other statistical analyses were performed using the Statistical Package for the Social Sciences version 21.0 for Windows (IBM Corp., Armonk, NY, USA). Statistical significance was defined as P < 0.05.

## Results

Initially 54 patients agreed to participate in the study, but four (2 narrow angle glaucoma, 2 high myopia) did not meet the inclusion criteria. Additionally, eight eyes were excluded because of poor-quality SS-OCT images (3), motion artifacts (3), or off-center fovea (2) and three subjects who failed to complete more than 4 sessions of testing were excluded from further analysis. Thus, finally, there were 39 eyes (20 POAG eyes of 20 patients and 19 healthy eyes of 19 subjects) available for analysis.

Patients were allowed to continue on their prescribed topical anti-glaucoma medications. At the time of imaging, 18 of the 20 patients (90.0%) in the POAG group were on topical antiglaucoma medications whether monotherapy or combination (prostaglandin analogues, 17 [85.0%]; β-blockers, 14 [70.0%]; carbonic anhydrase inhibitors, 12 [60.0%]; α-adrenergics, 7 [35.0%]). Two patients (10.0%) in the POAG group had undergone laser trabeculoplasty on the study eye. Five POAG patient (25.0%) and four healthy subjects (21.1%) had undergone remote cataract surgery on the study eye.

The baseline demographics are summarized in Table 1. No significant inter-group difference was found in age, sex, body-mass index, diabetes mellitus, hypertension, spherical equivalent, axial length, central corneal thickness, or baseline IOP (all P > 0.05).

**Table 1:** Demographics and clinical characteristics of healthy subjects and POAG.

	Healthy group (N = 19)	POAG group (N = 20)	*P*-value
Age (year)	51.68 ± 18.04	54.15 ± 16.14	0.655^a^
Sex (Male : Female)	11 : 8	9 : 11	0.266^b^
Body-mass index (Kg/m^2^)	24.10 ± 2.10	23.90 ± 3.51	0.836^a^
Diabetes mellitus, n (%)	3 (15.8%)	3 (15.0%)	0.878^b^
Hypertension, n (%)	5 (26.4%)	6 (30.0%)	0.945^b^
Spherical equivalent (Diopters)	-1.38 ± 2.29	-2.61 ± 3.37	0.193^a^
Axial length (mm)	24.10 ± 1.21	24.35± 1.30	0.597^a^
Central corneal thickness (μm)	529.00 ± 28.23	535.77± 44.90	0.658^a^
Baseline IOP (mmHg)	14.53 ± 1.64	14.97 ± 2.42	0.469^a^
Baseline vertical cup-to-disc ratio	0.43 ± 0.14	0.78 ± 0.98	<0.001^a^
Mean cpRNFL thickness	95.73 ± 11.16	66.50 ± 13.69	<0.001^a^
Mean deviation (dB)	0.17 ± 4.05	-8.94± 8.47	<0.001^a^

^a^Student t-test

^b^Chi-square test

cpRNFL, circumpapillary retinal nerve fiber layer

### Assessment of repeatability and reproducibility

The repeatability and reproducibility of RVD obtained by SS-OCTA were excellent for the POAG patients and healthy subjects (Table 2). The ICCs for intra-visit repeatability of peripapillary RVD showed excellent reliability (0.895–0.967), as did those for macular RVD (0.833–0.943). The ICCs for inter-visit reproducibility of peripapillary RVD ranged from 0.882 to 0.986, and for macular RVD, from 0.755 to 0.935. Also, there was no difference of inter- or intra-visit reliability between the healthy and POAG groups (all P < 0.05).

**Table 2:** Intra-visit repeatability and inter-visit reproducibility of retinal vessel density in healthy and POAG groups.

	Intra-visit repeatability ICC (lower-upper 95% CI)				Inter-visit reproducibility ICC (lower-upper 95% CI)			
	Total subjects	Healthy subjects	POAG	*P*-value^a^	Total subjects	Healthy subjects	POAG	*P*-value^a^
Peripapillary RVD								
Disc	0.967 (0.878–0.994)	0.944	0.987	0.664	0.975 (0.871–0.975)	0.949	0.990	0.842
pSuperior	0.890 (0.691–0.980)	0.821	0.937	0.165	0.986 (0.929–0.998)	0.865	0.995	0.265
pInferior	0.911 (0.789–0.989)	0.869	0.943	0.526	0.948 (0.737–0.994)	0.914	0.967	0.456
pNasal	0.895 (0.771–0.988)	0.910	0.854	0.409	0.974 (0.869–0.997)	0.940	0.984	0.833
pTemporal	0. 889 (0.740–0.982)	0.903	0.842	0.516	0.882 (0.704–0.987)	0.866	0.942	0.274
Macular RVD								
Fovea	0.852 (0.699–0.968)	0.921	0.790	0.165	0.900 (0.797–0.989)	0.914	0.878	0.598
mSuperior	0.943(0.714–0.994)	0.908	0.967	0.855	0.755 (0.671–0.947)	0.714	0.827	0.206
mInferior	0.908 (0.689–0.980)	0.874	0.900	0.275	0.874 (0.746–0.986)	0.884	0.870	0.258
mNasal	0.907 (0.709–0.981)	0.914	0.899	0.627	0.911 (0.705–0.982)	0.963	0.853	0.408
mTemporal	0.833 (0.637–0.964)	0.842	0.935	0.478	0.935 (0.764–0.990)	0.958	0.914	0.511

^a^As analyzed by repeated-measures ANOVA between Healthy and POAG groups, respectively.

ICC, intraclass correlation coefficient; CI, coefficient interval; RVD, retinal vessel density.

### Diurnal distributions of RVD and hemodynamic parameters

The RVD of the peripapillary (disc, superior, inferior, temporal, nasal) and macular (fovea, superior, inferior, temporal, nasal) areas were assessed in detail (Table 3). In the healthy group, there was no significant diurnal variation in any peripapillary or macular area, whereas the POAG group showed sizeable variation in the temporal area of the parafoveal region (P = 0.015). Among the hemodynamic parameters, SBP, DBP, and MOPP of POAG showed significant change on diurnal fluctuation (P = <0.001, <0.001, and 0.006, respectively).

**Table 3:** Diurnal distributions of retinal vessel density (%) and hemodynamic parameters for each measurement session with ANOVA P values.

	Healthy group (N = 19)						POAG group (N = 20)					
	8AM	11AM	2PM	5PM	8PM	^a^*P-* value	8AM	11AM	2PM	5PM	8PM	^a^*P-* value
Peripapillary RVD (avg.)	54.70±7.49	55.05±6.88	54.73±6.42	54.66±6.96	55.32±6.26	0.302	44.89±10.20	45.08±9.54	44.53±9.58	44.70±9.62	43.43±9.30	0.291
Disc head	41.56±11.10	41.37±11.22	43.23±6.16	39.81±9.27	39.19±7.92	0.355	26.39±15.82	25.51±16.71	19.05±15.56	28.49±14.94	23.71±10.55	0.239
pSuperior	65.18±5.42	65.47±3.16	63.58±3.79	65.63±3.29	66.39±2.87	0.560	55.18±5.74	54.60±4.97	53.52±8.06	51.48±6.81	52.03±7.41	0.440
pInferior	62.67±6.95	65.13±2.60	61.53±3.77	67.18±5.46	69.28±4.12	0.294	47.19±10.71	49.29±4.18	49.79±4.40	49.78±5.14	48.32±6.96	0.688
pTemporal	57.46±10.02	55.65±8.37	57.43±9.38	53.64±7.75	53.46±6.12	0.187	49.76±6.01	48.44±11.24	49.89±11.41	47.15±11.85	48.75±6.36	0.503
pNasal	46.62±3.98	47.64±9.06	47.89±9.02	47.06±9.01	48.27±10.25	0.842	45.94±12.74	47.58±10.62	50.39±8.49	46.62±9.38	44.36±15.23	0.213
Macular RVD (avg.)	43.13±2.49	42.21±3.20	43.95±3.65	43.31±2.50	44.06±2.60	0.244	41.24±4.79	42.48±5.64	41.03±5.29	40.93±4.79	39.96±5.10	0.294
Fovea	13.93±2.25	11.78±6.01	17.52±5.18	14.17±1.78	15.33±2.40	0.423	14.97±5.18	14.92±6.17	14.14±6.05	14.54±4.59	14.30±6.31	0.554
mSuperior	51.01±5.09	50.94±2.59	48.23±3.30	53.18±1.77	52.36±4.48	0.266	51.22±2.77	52.20±6.11	50.47±3.47	50.75±2.94	49.08±3.79	0.292
mInferior	52.84±2.88	52.60±1.90	56.72±5.36	51.54±4.43	52.68±3.26	0.256	46.44±8.52	48.46±7.01	49.69±6.40	47.67±7.17	48.32±6.86	0.180
mTemporal	48.44±1.13	48.62±4.27	47.10±2.13	47.06±2.25	49.68±1.19	0.493	45.36±2.90	47.57±4.51	45.01±2.88	44.71±3.24	42.95±3.61	**0.015**
mNasal	49.44±1.09	47.12±1.21	50.17±2.26	50.58±2.28	50.26±1.69	0.186	48.19±4.58	49.26±4.40	45.82±7.63	46.96±5.99	45.14±4.93	0.065
SBP (mmHg)	125.52±12.53	124.47±13.88	123.73±13.36	122.10±12.35	122.89±12.67	0.658	133.45±14.31	124.10±15.18	127.50±14.93	126.15±12.07	120.65±11.16	**<0.001**
DBP(mmHg)	81.63±12.07	82.10±11.17	77.63±12.90	80.00±10.25	79.63±9.58	0.296	78.85±9.75	75.30±11.84	76.20±7.33	74.30±9.28	66.40±5.28	**<0.001**
IOP (mmHg)	14.36±1.57	14.31±1.73	14.36 ±1.97	14.47±2.38	15.15±2.14	0.291	15.05±2.21	15.05±2.35	14.55±2.23	15.09±2.91	15.11±2.93	0.302
MOPP (mmHg)	55.35±8.05	55.27±8.31	52.17±8.04	53.91±7.18	53.80±8.56	0.244	54.66±7.17	51.01±7.75	52.50±5.92	50.98±6.53	47.36±4.53	**0.006**

^a^Repeated-measures ANOVA; bolded values represent significance, P< 0.05

RVD, retinal vessel density; avg., average; SBP, systolic blood pressure; DBP, diastolic blood pressure; IOP, intraocular pressure; MOPP, mean ocular perfusion pressure.

### Magnitudes of diurnal variation of RVD and hemodynamic parameters

To determine the degree of diurnal variation, the maximal change (maximum—minimum value among 5 measurements) of RVD and hemodynamic parameters was analyzed (Table 4). The magnitude of average diurnal peripapillary and macular RVD variations in POAG (9.71 ± 7.04 and 7.22 ± 4.73%) were more variable than in the healthy group (5.73 ± 3.85 and 5.51 ± 3.45%) (P = 0.013 and 0.042). In detail, RVD’s diurnal change of optic disc head, superior peripapillary, and inferior parafoveal areas in the POAG eyes were larger than in the healthy eyes (P = 0.005, 0.006, and 0.034, respectively). Similarly, among the hemodynamic parameters, the maximal changes of IOP, DBP and MOPP in POAG were greater than in the healthy group (P = 0.003, 0.014 and 0.039).

**Table 4:** The magnitude (maximum diurnal changes [ΔMax.]) of diurnal variation of retinal vessel density and hemodynamic parameters between healthy subjects and POAG.

	Healthy subjects (N = 19)	POAG (N = 20)	*P*-value^a^
ΔMax.in peripapillary RVD (%)			
Average	5.73±3.85	9.71±7.04	**0.013**
Disc head	6.32±3.74	13.02±9.18	**0.005**
pSuperior	5.01±2.59	8.30±4.16	**0.006**
pInferior	5.18±4.77	8.07±5.61	0.091
pTemporal	5.96±4.36	8.59±4.85	0.084
pNasal	6.19±3.81	10.55±11.40	0.119
ΔMax.in macular RVD (%)			
Average	5.51±3.45	7.22±4.73	**0.042**
Fovea	5.10±3.90	4.37±3.87	0.559
mSuperior	5.71±3.73	7.94±3.77	0.051
mInferior	5.98±3.18	8.92±4.95	**0.034**
mTemporal	5.66±3.15	6.72±6.77	0.540
mNasal	5.28±3.28	7.13±4.28	0.139
ΔMax.in hemodynamics (mmHg)			
SBP	17.26±9.45	22.25±8.34	0.089
DBP	14.73±8.94	21.40±7.10	**0.014**
IOP	2.84±1.01	3.95±1.19	**0.003**
MOPP	9.80±5.70	13.41±4.84	**0.039**

^a^Independent t-test, bolded values represent significant, *P*< 0.05

RVD, retinal vessel density; SBP, systolic blood pressure; DBP, diastolic blood pressure; IOP, intraocular pressure; MOPP, mean ocular perfusion pressure.

### Patterns of diurnal variation of RVD and hemodynamic parameters between healthy subjects and POAG

**Fig 2:**
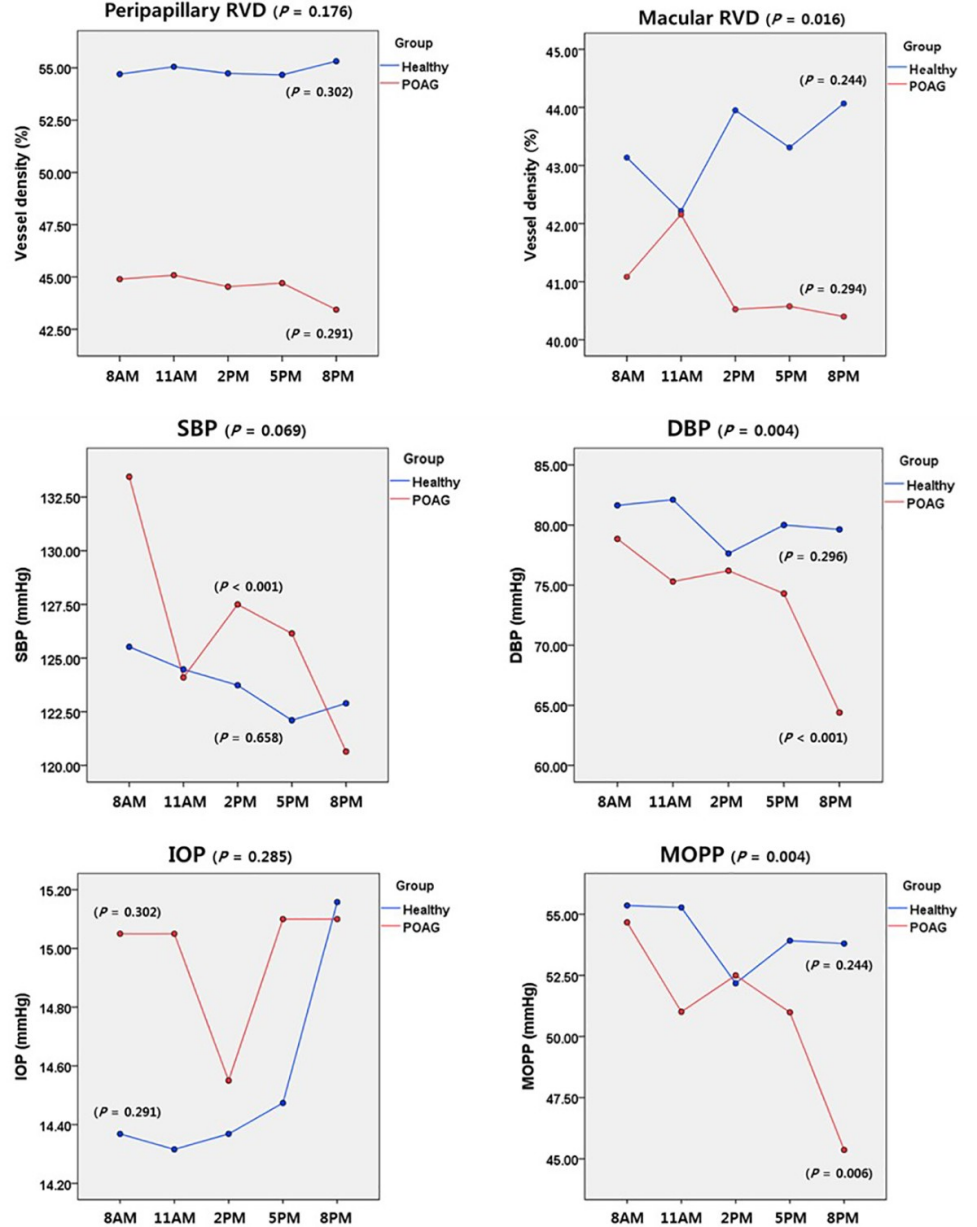
Patterns of diurnal variation of RVD and hemodynamic parameters in healthy subjects and POAG patients. The average RVDs in the peripapillary (optic disc head, superior, inferior, temporal, nasal) and macular (fovea, superior, inferior, temporal, nasal) areas are shown at the top. SBP, DBP, IOP, and MOPP were recorded every 3 hours from 8 a.m. to 8 p.m. The RVD and hemodynamic variations in each group were analyzed by ANOVA, and the inter-group comparison was assessed by a linear mixed model.

The patterns of diurnal changes of RVD, BP, IOP, and MOPP in each group were examined, and the intra- and inter-group ANOVA P values were assessed together (Fig 2). There was no significant pattern of diurnal peripapillary or macular RVD change in either group. However, a comparative analysis between the two groups showed a significant difference in the diurnal pattern of macular RVD change (P = 0.016). Specifically, the macular RVD of the healthy group had increased to 44.12 ± 2.95% at 8 p.m., whereas that of the POAG group had decreased to 40.41 ± 2.54% by that time (P = 0.047, Table 5). As for the hemodynamic parameters, the POAG group had the peak values at 8 a.m. (DBP 78.85 ± 9.75 and MOPP 54.66 ± 7.17mmHg) and the trough values at 8 p.m. (74.30 ± 9.28 and 50.98 ± 6.53mmHg, P = 0.004 and 0.004, respectively). In addition, among the hemodynamic parameters, MOPP showed a significant correlation with both peripapillary and macular RVD (see the online S2 Table).

**Table 5:** Post hoc analysis of macular RVD, DBP, and MOPP in healthy subjects and POAG.

	Healthy subjects (N = 19)	POAG (N = 20)	*P*-value^a^
Average macular RVD (%)			
8 AM	43.05 ± 3.22	41.28 ± 2.53	0.258
11 AM	42.26 ± 1.36	42.20 ± 3.04	0.830
2 PM	43.96 ± 3.02	42.15 ± 2.29	0.068
5 PM	43.01 ± 2.44	41.59 ± 3.06	0.184
8 PM	44.12 ± 2.95	40.41 ± 2.54	**0.047**
DBP (mmHg)			
8 AM	81.63 ± 12.07	78.85 ± 9.75	0.435
11 AM	82.10 ± 11.17	75.30 ± 11.84	0.073
2 PM	77.63 ± 12.90	76.20 ± 7.33	0.676
5 PM	80.00 ± 10.25	74.30 ± 9.28	0.078
8 PM	79.63 ± 9.58	66.40 ± 5.28	**<0.001**
MOPP (mmHg)			
8 AM	55.35 ± 8.05	54.66 ± 7.17	0.779
11 AM	55.27 ± 8.31	51.01 ± 7.75	0.106
2 PM	52.17 ± 8.04	52.50 ± 5.92	0.887
5 PM	53.91 ± 7.18	50.98 ± 6.53	0.192
8 PM	53.80 ± 8.56	47.36 ± 4.53	**0.001**

^a^Independent t-test; bolded values represent significance, *P*< 0.05

RVD, retinal vessel density; DBP, diastolic blood pressure; MOPP, mean ocular perfusion pressure

### Representative cases

**Fig 3:**
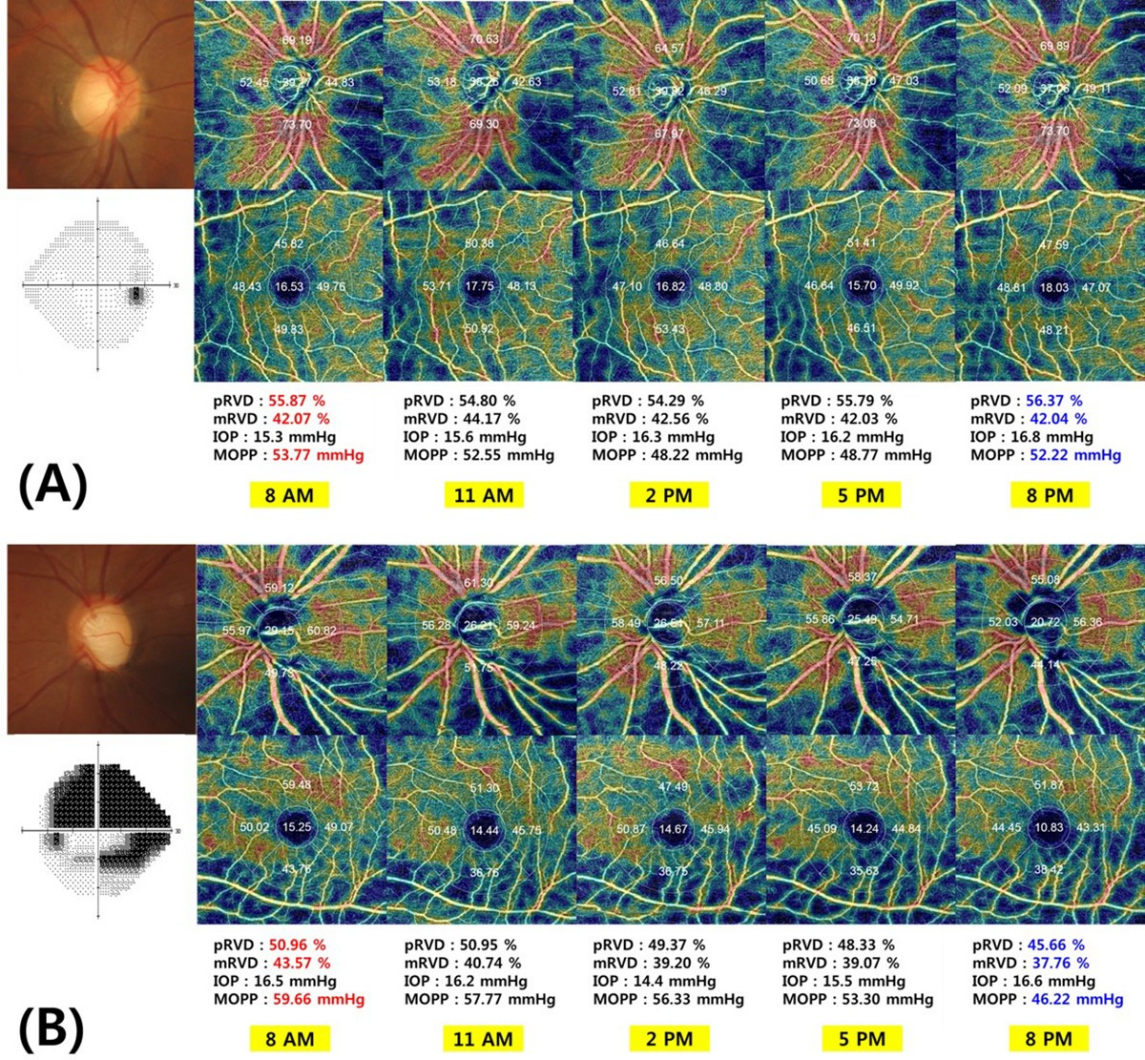
Representative cases of diurnal variation of peripapillary retinal vessel density (pRVD) and macular retinal vessel density (mRVD). **(A)** A 44-year-old woman enrolled among the healthy subjects demonstrated an average pRVD of 55.87% and an average mRVD of 42.07% at 8 a.m. At 2 p.m., MOPP was the lowest, 48.22mmHg, and pRVD, 55.79%, was slightly decreased compared with 8 a.m., but at 8 p.m., MOPP had increased again to 52.22mmHg,with elevated pRVD and mRVD of 56.37 and 42.02%, respectively.**(B)** A 46-year-oldwoman with POAG (VF MD, -19.72 dB) had a MOPP of 59.66mmHg, an average pRVD of 50.96%, and an average mRVD of 43.57%, all of which were the highest daily values. At 5 p.m., the pRVD and mRVD had decreased to 48.33 and 39.07%, respectively, accompanied by a MOPP of 53.30 mmHg. However, at 8 p.m., all had decreased further, to 45.66, 37.76, and 46.22% for MOPP, pRVD, and mRVD, respectively.

Fig 3 depicts representative cases showing diurnal variation of RVD inthe (A) healthy and (B) POAG groups.

## Discussion

In the present study, the diurnal variation of RVD was compared between POAG patients and healthy subjects. We found that (1) the diurnal fluctuations of macular RVD, DBP, and MOPP were greater in the POAG group and (2) the patterns of diurnal variation differed, where disparities were greatest in the evening time.

The degrees of diurnal change of macular RVD accompanied by DBP and MOPP were greater in the POAG patients than in the healthy subjects. Previous studies have identified increased variability of MOPP as a risk factor for glaucoma [26–28]; similarly, the present study observed significant variations of systemic hemodynamics and ocular hemodynamic changes at the level of the retinal blood vessels simultaneously in POAG. Another possible explanation is the effect of compensation mechanisms. In a normal group, there is autoregulation to compensate for variation of MOPP, and therefore, there is no significant fluctuation of retinal circulation. On the other hand, it could be deduced that this compensation mechanism does not work properly in POAG groups [27, 29].

Recently, Mansouri et al.[15] reported diurnal variation of peripapillary and macular RVD in healthy subjects and open-angle glaucoma. The results demonstrated that the diurnal changes in OCTA-measured RVD in glaucoma patients were small and clinically insignificant. This discrepancy from our results appears to be due to the difference of study design. In our study, the macular RVD difference between the two groups was prominent in the evening, and its possible associated factors were DBP and MOPP, which also decreased in the evening. However, in Mansouri's study, the RVD in the evening was not measured (it was measured only until 5 p.m.), and neither the BP nor the MOPP was evaluated.

The choroid is a vascular-rich structure the thickness of which changes dynamically depending on its hemodynamic physiology [30–32]. Accordingly, diurnal fluctuation of choroidal thickness provides us with good indications for understanding of retinal vascular changes. Previous studies on diurnal variation of choroidal thickness in healthy subjects showed that the thickest choroid in the morning thinned in the afternoon and thickened again in the evening [22, 33]. Based on these results, we could infer increasing RVD among the healthy group in the evening. On the other hand, the reduced RVD of POAG in the afternoon did not increase again in the evening. These findings could be closely related to changes in BP and MOPP as well as large fluctuation of RVD in POAG. As is well known, nocturnal reduction of DBP and MOPP in glaucoma patients has been confirmed in previous studies [14, 30, 31].

In this study, diurnal variation of DBP except SBP in POAG group was larger than that in healthy subjects. Although limited, a possible explanation could be inferred from previous studies. There are several reports that low systemic DBP is associated with glaucoma progression [34, 35]. In addition, low DBP level is also associated with organ damage (e.g., cardiac ischemic damage, micro-albuminuria) [36, 37]. Based on these findings, although additional analysis and evidence will be needed, it can be deduced that DBP fluctuation plays a more important role in end organ damage, including damage to the optic nerve head.

### Study limitations

Several points need to be considered when interpreting the results of the current study. First, diurnal variation was measured in patients who were taking ocular hypotensive medication. According to previous reports, ocular hypotensive medication reduces diurnal fluctuation [38, 39]. Therefore, the actual severity of diurnal RVD variation might have been underestimated in our study. Second, RVD and hemodynamics were assessed only during the daytime, not at midnight. Therefore, the significant pattern of RVD variation that we observed might not reflect whole diurnal variation. In the near future, additional studies are needed to analyze RVD at midnight and dawn based on 24-hour monitoring of BP and IOP. Lastly, in the peripapillary RVD, the superior sector showed a significant difference; however, in the macular RVD, the inferior sector showed a difference between healthy subjects and POAG. Since the superior and inferior peripapillary RNFL are connected to the superior and inferior sectors of the macula, respectively, the results of this study could be questionable. Considering that early RNFL defect usually develops in the superotemporal or inferotemporal area, the mapping established for sectoral RVD analysis in this study was not ideal for reflecting the actual pattern of glaucomatous damage. As a result, sector discordances between peripapillary and macular RVD might have occurred. Therefore, the evaluated area of peripapillary RVD or macular RVD needs to be subdivided specifically (superotemporal, superonasal, inferotemporal, etc.).

In conclusion, in POAG eyes, diurnal change of IOP, MOPP and RVD was significantly greater than in the healthy eyes. These findings suggest that diurnal RVD changes might reflect the hemodynamic variation of POAG. Additionally, our results suggest that when performing RVD measurement, it is necessary to consider that diurnal variation occurs at specific times, and that therefore, for accurate analysis, measurement times should be consistent. We hope that our paper will be a helpful reference for comparative analysis of RVD in future OCTA studies.

## Supporting information

S1 Table Diurnal distributions of retinal vessel density. (XLSX)

S2 Table Correlation between fluctuation of RVD and BP, IOP or MOPP using mixed effect model. (PDF)
