# Supplementary material for: Diurnal change of retinal vessel density and mean ocular perfusion pressure in patients with open-angle glaucoma
Source: PLoS One. 2019 Apr 26;14(4):e0215684. doi: 10.1371/journal.pone.0215684 (PMC6485647; doi:10.1371/journal.pone.0215684)
Supplement: S2 Table — (PDF) [file pone.0215684.s002.pdf]

Supplementary Table 2. Correlation between fluctuation of RVD and BP, IOP or MOPP in total subjects using mixed effect model

Correlation using mixed effect model			
Variables		Average peripapillary RVD	Average macular RVD
SBP	β	0.041	-0.069
	P value[a]	0.102	0.078
DBP	β	0.175	0.118
	P value[a]	0.116	0.290
IOP	β	-0.257	-0.047
	P value[a]	**0.020**	0.635
MOPP	β	0.060	0.351
	P value[a]	**<0.001**	**<0.001**

[a]Linear mixed model; bolded values represent significance, $P < 0.05$

RVD, retinal vessel density; SBP, systolic blood pressure; DBP, diastolic blood pressure; IOP, intraocular pressure, MOPP, mean ocular perfusion pressure
